# A Computational Framework for Automated Reconstruction and Analysis of Dynamic Consent Interaction

**DOI:** 10.3390/s26144510

**Published:** 2026-07-16

**Authors:** Maysoon Abulkhair

**Affiliations:** Information Technology Department, Faculty of Computing and Information Technology, King Abdulaziz University, Jeddah 21589, Saudi Arabia; mabualkhair@kau.edu.sa

**Keywords:** dynamic consent ecosystems, Adaptive Cognitive Load Dark Patterns (ACL-DP), Consent Management Platforms (CMP), computational sensing, usable privacy, privacy engineering, Human–Computer Interaction (HCI), behavioral privacy analytics

## Abstract

Dynamic consent ecosystems have become increasingly complex due to the widespread adoption of Consent Management Platforms (CMPs), multi-layer preference interfaces, asynchronous rendering architectures, and adaptive interaction workflows. Existing privacy-auditing approaches primarily rely on static interface inspection and therefore provide limited support for reconstructing and evaluating dynamic consent interactions. To address these limitations, this study proposes a computational measurement framework for the automated reconstruction and analysis of dynamic consent ecosystems. The framework integrates five computational layers for browser-based acquisition, interaction sensing, multi-layer synchronization, consent-state verification, and Adaptive Cognitive Load Dark Pattern (ACL-DP) operationalization. The proposed methodology combines asynchronous browser automation, interaction workflow reconstruction, multi-source evidence synchronization, backend consent verification, and rule-based mechanism scoring to transform complex consent interactions into reproducible quantitative representations. Evaluation across 18,665 consent ecosystems generated 59 synchronized variables spanning interface, interaction, textual, and consent-state dimensions. Workflow reconstruction successfully recovered interaction trajectories for 99.6% of observable consent environments, while backend verification identified consent mismatches in 77.6% of environments with complete frontend–backend aligned evidence, revealing substantial divergence between observable consent decisions and backend consent behavior. The ACL-DP framework operationalizes four mechanism families, Effort Engineering, Attention Engineering, Cognitive Load Amplification, and Algorithmic Adaptivity, the latter capturing observable runtime, session-dependent, context-sensitive, and backend-mediated variation in consent behavior. The results revealed persistent procedural and attentional asymmetries, recurrent hidden rejection mechanisms, and widespread frontend–backend consent inconsistencies, with Effort Engineering emerging as the dominant manipulation strategy. Validation through reproducibility analysis, sensitivity analysis, statistical uncertainty assessment, and a human benchmark of 100 independently annotated websites demonstrated high inter-run consistency and moderate-to-substantial inter-rater agreement, supporting the framework’s reliability and validity. This work provides a reproducible foundation for large-scale privacy interaction analysis and evidence-based evaluation of dynamic consent ecosystems.

## 1. Introduction

The rapid expansion of web-based digital services has transformed online privacy management into a complex socio-technical ecosystem that involves users, tracking infrastructures, regulatory frameworks, and automated consent management technologies. Cookie-consent interfaces have emerged as a central mechanism for regulating user permissions and personal data processing under privacy regulations such as the General Data Protection Regulation (GDPR), which requires explicit, informed, and freely given consent for personal data collection and processing activities. To satisfy these regulatory requirements, organizations increasingly deploy Consent Management Platforms (CMPs) that standardize consent acquisition procedures and synchronize tracking permissions across heterogeneous web environments [1]. CMPs provide structured infrastructures to manage user consent preferences while supporting regulatory compliance objectives and user interaction management processes [2].

Despite their widespread adoption, the increasing complexity of consent ecosystems presents substantial challenges for automated measurement, monitoring, and large-scale analysis [3]. Contemporary consent interfaces frequently rely on asynchronous JavaScript execution, dynamically generated content, multi-layer interaction workflows, embedded third-party components, and adaptive rendering strategies. These characteristics limit the effectiveness of conventional static crawling and extraction approaches, which often fail to capture the dynamic, procedural, and context-dependent characteristics of consent interactions. Consequently, existing computational studies have largely focused on isolated interface attributes or static extraction methods, providing limited support for synchronized multi-layer analysis of consent ecosystems at scale.

Recent research in Human–Computer Interaction (HCI), privacy engineering, and behavioral decision-making demonstrates that consent interfaces function not only as informational mechanisms but also as behavioral choice architectures that influence user decision-making processes. Empirical studies have shown that interface asymmetries, hidden rejection pathways, visually prioritized acceptance options, procedural complexity, and cognitive burden can systematically affect user behavior, frequently favoring service-provider objectives over user autonomy and informed decision-making [4]. Although these studies have substantially advanced understanding of manipulative consent practices, existing computational approaches remain largely descriptive and do not fully operationalize the behavioral mechanisms underlying consent interactions within scalable, reproducible, and empirically validated analytical infrastructures.

To address these limitations, this study proposes a scalable multi-layer computational measurement framework for automated acquisition, reconstruction, synchronization, and analysis of dynamic consent ecosystems. The proposed framework integrates distributed browser acquisition, interaction sensing, multi-layer synchronization, consent-state verification, and Adaptive Cognitive Load Dark Pattern (ACL-DP) operationalization to enable large-scale empirical investigation of contemporary consent interfaces. Because each analytical stage imposes different technical observability and reconstruction requirements, the framework explicitly differentiates among multiple analytical populations, including acquired environments, successfully rendered environments, dynamic workflow reconstruction records, backend verification records, and measurable synchronization cases. In particular, frontend–backend synchronization and consent-state verification require progressively stricter evidentiary conditions, resulting in a smaller subset of environments for which complete synchronization among observable user interactions, browser storage states, network activity, and backend consent persistence can be reliably reconstructed.

Beyond methodological development, study emphasizes rigorous empirical validation through human benchmark annotation, repeated-run reproducibility assessment, benchmark reconstruction consistency evaluation, and statistical robustness analysis. ACL-DP refers to interface mechanisms that intentionally increase, exploit or increase the cognitive burden to influence decision-making processes in ways that favor platform objectives, often at the expense of user autonomy, informed consent and privacy preferences [5]. To operationalize these mechanisms, the ACL-DP framework employs an explicitly defined weighting and aggregation strategy to construct composite manipulation indicators and evaluates their robustness through sensitivity analysis.

Algorithmic Adaptivity (AA) is defined as the observed capacity of consent ecosystems to exhibit a runtime, session-dependent, context-sensitive, storage-dependent or backend-mediated variation in consent behavior. Examples include dynamic interface rendering, geolocation-sensitive configurations, session-state dependencies, backend consent propagation, iframe-mediated interaction workflows, and dynamic Layer-2 preference reconstruction. Importantly, AA characterizes observable adaptive properties of consent ecosystems rather than adaptive behavior of the ACL-DP framework itself. The proposed framework does not employ artificial intelligence, machine learning, predictive analytics, reinforcement learning, or adaptive optimization. Instead, it relies on browser automation, workflow reconstruction, evidence synchronization, backend verification, and rule-based computational measurement procedures.

Because large-scale consent measurement is susceptible to detection failures, iframe embedding, multilingual variation, asynchronous rendering behavior, and anti-automation mechanisms, the proposed framework incorporates explicit quality-control procedures for consent detection, interaction reconstruction, synchronization verification, and benchmark validation. Furthermore, because composite manipulation indicators can be sensitive to feature aggregation strategies and threshold definitions, the ACL-DP framework explicitly specifies its scoring assumptions and evaluates their robustness through sensitivity analyzes.

This methodological design enables scalable and reproducible measurement of procedural asymmetry, attentional prioritization, cognitive burden, adaptive consent behavior, consent-state inconsistencies, hidden rejection mechanisms, and acceptance-oriented interaction pathways across heterogeneous web environments. In addition, the framework supports methodological reproducibility through repeated pipeline execution, benchmark reconstruction consistency assessment, and independent human annotation agreement analysis.

The study is guided by the following research questions:

- RQ1: How can dynamic consent interaction ecosystems be represented through a unified multi-layer computational sensing architecture that integrates interface structure, interaction behavior, textual complexity and consent-state outcomes?
- RQ2: How can multi-layer consent interactions be automatically reconstructed and synchronized across heterogeneous web environments at scale?
- RQ3: How can the results of the computational measurements be operationalized as measurable indicators of the ACL-DP mechanism for systematic interaction analysis?
- RQ4: How can the proposed computational measurement framework ensure scalability, reproducibility, robustness, and validation consistency across dynamic consent ecosystems?

RQ4 investigates whether the proposed ACL-DP framework achieves scalability, reproducibility, and robustness under large-scale web measurement conditions through repeated pipeline execution, benchmark reconstruction consistency assessment, inter-run agreement analysis, sensitivity analysis, and independent human annotation validation. The main contributions of this study are summarized as follows:

1. A scalable multi-layer computational measurement framework for the automated monitoring and reconstruction of dynamic consent ecosystems;
2. A distributed interaction-sensing architecture capable of reconstructing synchronized Layer-1 and Layer-2 consent workflows across heterogeneous CMP environments;
3. A unified analytical representation integrating interface, interaction, textual, and consent-state evidence layers within a standardized computational framework;
4. A computational operationalization methodology for ACL-DP mechanisms using measurable interface, interaction and behavioral indicators;
5. A reproducible large-scale computational infrastructure supporting distributed execution, robustness evaluation, synchronized dataset generation, benchmark validation, and empirical analysis of consent interaction ecosystems.

## 2. Literature Review

Research on cookie consent interfaces has progressively evolved from small-scale compliance observations to large-scale computational monitoring systems and structured analytical datasets capable of supporting automated web measurement, behavioral interaction analysis, and mechanism-level computational interpretation.

### 2.1. Evolution of Cookie Consent Research

Cookie consent research has evolved from a narrow compliance-oriented concern into a broader HCI and usable privacy research domain. Early work treated cookie notices mainly as regulatory artifacts introduced after the GDPR, the ePrivacy Directive, and CCPA. However, subsequent studies demonstrated that the mere presence of a consent banner does not guarantee meaningful, informed, or usable consent. Habib et al. showed that cookie consent interfaces impose a substantial user burden and that interface prominence, inline controls, and persistent preference mechanisms significantly influence user awareness and decision-making [2]. Sanchez-Rola et al. reported similar concerns, analyzing 2000 high-traffic websites, and found that tracking activities frequently remained active despite the presence of consent notices, while many websites provided misleading or ineffective opt-out mechanisms [6]. These findings established that consent analysis must extend beyond visible interface elements to include tracking behavior and post-interaction consent states.

### 2.2. Large-Scale Computational Monitoring

A major methodological development has been the transition from small-scale manual inspection to large-scale computational monitoring. Nouwens et  al. developed a computational audit framework to extract consent interfaces from the five most widely deployed CMPs on the top 10,000 UK websites, generating a dataset of 680 consent interface designs and demonstrating that hidden rejection pathways and absent first-layer opt-out controls significantly increased acceptance behavior [7]. Matte et al. subsequently extended consent auditing by analyzing 28,257 websites and identifying 1426 websites implementing the IAB Transparency and Consent Framework (TCF), revealing widespread inconsistencies between visible consent choices and backend consent signals [8]. These studies established the importance of integrating observations from the interface-layer with consent-state monitoring at the browser-level and the network-level .

### 2.3. Behavioral and Cognitive Perspectives

Behavioral interaction analysis has emerged as a central research direction in consent ecosystem evaluation. Jha et al. analyzed more than four million real-world consent interactions across 434 websites served by a global CMP infrastructure. Their findings showed that multi-step rejection pathways substantially reduced opt-out behavior, while introducing a one-click rejection option significantly increased refusal rates [9]. These observations align with theories of bounded rationality and cognitive load, suggesting that procedural friction can systematically influence privacy decisions. Habib et al. similarly observed that users frequently select the least effortful option when confronted with repeated consent decisions, highlighting the cumulative burden imposed by modern consent interfaces [2].

### 2.4. Textual and Semantic Analysis

Textual and semantic analysis has become increasingly important because consent depends not only on interface structure but also on the clarity, specificity, and interpretability of language. Santos et al. collected approximately 1300 cookie banners from popular European websites and manually annotated nearly 400 banners according to legal-transparency criteria. Their results showed that many interfaces relied on vague language, excessive jargon, and ambiguous framing strategies, indicating that consent comprehension is often constrained by disclosure complexity rather than interface structure alone [10]. Recent research has further demonstrated that large language models (LLMs) can identify and classify privacy disclosures with F1 scores exceeding 93%, enabling scalable semantic analysis of privacy communications.

### 2.5. Regional and Sector-Specific Studies

Regional studies demonstrate that consent practices vary substantially across legal, cultural, and sectoral contexts. Albesher et al. evaluated 144 high-traffic Saudi Arabian websites spanning the government, commerce, healthcare, finance, and education sectors and identified widespread problems involving limited user control, inaccessible rejection mechanisms, implied consent behaviors, and weak preference-management support [11]. Similarly, Srisumrith and Wisitpongphan analyzed 390 Thai websites using Playwright-based automated crawling and comprehensive manual validation, finding that automated auditing systems frequently missed region-specific interaction patterns, visual asymmetries, and manipulation strategies [12]. These studies highlight the importance of validation-aware computational infrastructures capable of supporting heterogeneous regulatory environments.

### 2.6. Methodological Standardization and Reproducibility

Methodological fragmentation remains a recurring challenge in consent research. Kretschmer et al. argued that standardized computational methodologies are essential to ensure comparability across web-measurement studies [13]. Existing investigations frequently differ in crawler configuration, rendering environments, interaction simulation logic, consent-state definitions, and validation procedures, making results difficult to reproduce and compare. The Thailand validation study further demonstrated that automated detection can substantially under-detect or over-detect dark-pattern categories without benchmark validation.

### 2.7. Composite Indicator Construction and Weighting Strategies

The construction of composite indicators requires careful consideration of weighting strategies because arbitrary weighting choices may introduce systematic bias. Existing composite-indicator frameworks commonly employ equal weighting when no validated theoretical or empirical evidence supports differential weighting among dimensions. Consequently, equal-weight aggregation remains a widely accepted baseline approach for multidimensional benchmark construction.

### 2.8. Mechanism-Level Operationalization

The literature increasingly moves beyond descriptive dark-pattern taxonomies toward the mechanism-level operationalization of consent manipulation strategies. Existing studies frequently operationalize Effort Engineering through indicators such as click asymmetry, hidden rejection pathways, multi-step opt-out processes, unequal navigation depth, and the absence of first-layer rejection controls, all of which increase the procedural burden during privacy decision-making [7,9]. Attention Engineering (AE) is commonly represented through visual salience asymmetries, an unequal button hierarchy, color contrast manipulation, visual prominence, and preselected options that steer user attention toward acceptance-oriented actions [7]. However, Cognitive Load Amplification (CL) remains less systematically operationalized and is typically inferred from dense preference structures, extensive disclosure text, layered navigation, semantic ambiguity, and information overload rather than being measured through dedicated computational indicators [2,10]. Similarly, algorithmic adaptation remains the least explored mechanism family, appearing primarily in studies of CMP behavior, consent-state regeneration, adaptive personalization, and tracking infrastructures rather than in synchronized consent interaction datasets [8]. Consequently, most existing frameworks focus on isolated indicators rather than synchronized computational representations capable of integrating interaction burden, attentional steering, textual complexity, and backend consent behavior. This limitation motivates the development of ACL-DP operationalization as a unified mechanism-aware analytical infrastructure.

### 2.9. Emerging Trends: AI-Driven Consent Analysis

AI-driven consent analysis is emerging in three directions. First, LLMs are used to analyze privacy policies and extract data practices at scale. Rodriguez et al. show that ChatGPT (gpt-3.5-turbo-0613 model) and Llama 2 can identify and categorize privacy policy disclosures with high performance, reducing the dependence on manual labor-intensive annotation [14]. Second, LLM-based systems such as CLEAR provide contextual privacy risk support by identifying sensitive user inputs, retrieving relevant privacy policy snippets, and generating just-in-time risk explanations for LLM applications. Third, LLMs are being explored as technical countermeasures against deceptive design. Schäfer et al. show that prompting GPT-4o to make manipulative HTML interfaces less manipulative produced less manipulative redesigns in 91% of deceptive elements after three iterations [15]. However, AI also introduces privacy risks. Work on LLM privacy emphasizes risks in training data, user prompts, model outputs, and LLM agents, while broader AI privacy scholarship argues that AI intensifies longstanding privacy problems by expanding inference, prediction, automation, and data generation. Therefore, AI-driven consent analysis should be framed as both an opportunity and a risk: AI can support scalable auditing, semantic interpretation, and interface correction, but it may also create new opacity, dependency, and governance challenges.

Recent advances in AI-assisted annotation have revitalized interest in large language models (LLMs) and agent-based systems as scalable alternatives to conventional human annotation workflows. Contemporary annotation research argues that LLM-powered agents can address longstanding challenges related to annotation cost, scalability, consistency, and the scarcity of domain expertise by combining language understanding, adaptive reasoning, and workflow orchestration capabilities. Agent-based annotation architectures further extend these capabilities by integrating autonomous decision-making, collaborative reasoning, self-reflection, and human-in-the-loop quality control mechanisms, thereby enabling efficient annotation workflows across heterogeneous data environments while preserving expert oversight for quality assurance and error correction tasks [16].

However, existing research also reveals important limitations. Although LLM-enhanced annotation systems demonstrate substantial improvements in throughput and operational efficiency, current evaluations remain concentrated on relatively structured tasks such as document classification, information extraction, medical coding, and sentiment analysis. Moreover, existing studies often rely on limited benchmark datasets and emphasize annotation performance metrics rather than broader concerns related to reproducibility, interpretability, regulatory validity, and behavioral reliability [16,17]. Consequently, despite growing evidence that LLM-generated annotations can achieve performance approaching human-level agreement under constrained conditions, questions remain regarding their applicability to complex human-centered domains characterized by ambiguity, contextual dependence, and behavioral interpretation.

These limitations are particularly salient in usable privacy and consent interface research. Unlike conventional annotation tasks, privacy consent interfaces constitute complex socio-technical systems in which visual presentation, interaction sequencing, cognitive burden, and contextual decision-making jointly influence user behavior. Previous Human-Computer Interaction (HCI) and usable privacy research has consistently demonstrated that interface characteristics such as effort asymmetry, hidden rejection mechanisms, button highlighting, visual salience, default configurations, and procedural complexity can systematically influence user consent decisions [18]. Furthermore, dual-process theories of cognition, bounded rationality, and digital nudging frameworks suggest that manipulative interface designs exploit users’ reliance on intuitive rather than deliberative decision-making processes, thereby affecting the autonomy and validity of consent choices [18].

In this context, it is essential to distinguish between two fundamentally different applications of LLMs: annotation and evaluation. Existing evidence suggests that LLMs are highly effective for annotation-oriented tasks, including extraction, classification, summarization, and reconstruction of privacy disclosures and consent interactions [14,16]. However, literature consistently cautions against treating LLMs as autonomous evaluators of legal compliance, user autonomy, or manipulative interface behavior. Research on AI-based consent management remains primarily concentrated within healthcare care and other narrowly defined domains, typically involving limited datasets, restricted evaluation settings, and substantial dependence on expert supervision to ensure reliability, transparency, and regulatory compliance [17].

In addition to methodological limitations, LLMs introduce significant privacy and regulatory challenges. Recent research demonstrates that contemporary models remain vulnerable to training-data memorization, information leakage, membership inference attacks, model inversion attacks, and accidental disclosure of sensitive information. More fundamentally, current LLM architectures exhibit structural incompatibilities with core privacy principles, including data minimization, purpose limitation, transparency, and the right to erasure established under the General Data Protection Regulation (GDPR) and related regulatory frameworks [19]. Existing mitigation approaches, including differential privacy, federated learning, retrieval-augmented generation, machine learning, and data sanitization, provide only partial solutions and do not satisfy all regulatory requirements simultaneously [19].

### 2.10. Synthesis and Research Motivation

The reviewed literature demonstrates three persistent limitations in existing consent analysis research. First, most studies analyze isolated dimensions of consent ecosystems without integrating interface behavior, interaction workflows, textual complexity, and consent-state outcomes within a unified analytical framework. Second, reproducibility and validation remain insufficiently addressed in large-scale consent measurement studies. Third, operationalization at the mechanism-level of manipulative consent strategies remains underdeveloped, particularly for adaptive and context-dependent consent behaviors.

To address these limitations, this study proposes the ACL-DP framework, which integrates browser-based acquisition, interaction reconstruction, multi-layer evidence alignment, backend consent verification, human validation, and mechanism-level behavioral analysis within a reproducible computational measurement architecture.

## 3. Methodology

This study proposes a computational framework for the acquisition, reconstruction, synchronization, verification, and analysis of dynamic consent interactions. The framework consists of five computational layers: browser-based acquisition, interaction sensing, multi-layer synchronization, consent-state verification, and ACL-DP operationalization.

The framework consists of five interconnected analytical layers:

1. Distributed browser-based acquisition;
2. Dynamic interaction sensing;
3. Multi-layer synchronization;
4. Consent-state verification;
5. ACL-DP operationalization.

These components collectively support the reconstruction and analysis of four complementary dimensions of consent ecosystems:

1. Interface structures;
2. Interaction behaviors;
3. Textual complexity;
4. Consent-state propagation.

**Figure 1:**
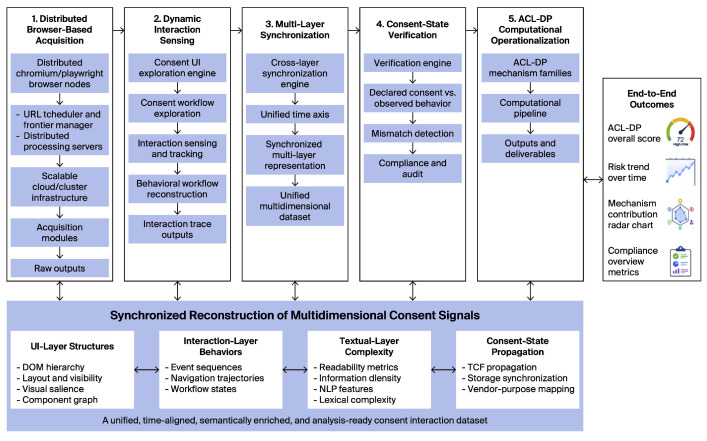
Computational Sensing and Automated Framework for dynamic consent interaction.

As illustrated in Figure 1, the proposed framework provides a reproducible analytical infrastructure for investigating dynamic consent ecosystems across heterogeneous CMP environments. Unlike existing consent auditing and dark-pattern detection approaches, the framework extends beyond consent banner detection by reconstructing complete interaction workflows, synchronizing frontend and backend consent evidence, operationalizing ACL-DP mechanisms, and including human benchmark validation and reproducibility assessment.

Consequently, the primary contribution of the proposed framework lies not in any individual methodological component but in the integration of interface presentation, interaction behavior, textual characteristics, consent-state propagation, and manipulation mechanisms within a unified computational measurement architecture.

### 3.1. Ethical and Responsible Web Measurement Procedures

The web measurement data collection process was conducted in accordance with established principles for responsible web measurement research. Data collection was restricted to publicly accessible web content and did not involve user authentication, account creation, credential submission, or the collection of personal information. To minimize the operational impact on the target websites, the browser execution used conservative concurrency settings (maximum concurrency = 5), controlled timeout policies (45 s) and request-throttling mechanisms. These measures were designed to reduce the server load and prevent service interruption during automated interaction procedures. The browser environment was configured using a standardized Chromium execution profile with a consistent user-agent identifier.

### 3.2. Methodology–Implementation Alignment of the CSA Framework

The Computational Sensing and Automated (CSA) Framework operationalizes consent ecosystems as synchronized computational environments by integrating browser-based acquisition, interaction sensing, evidence synchronization, consent-state verification, and analytical measurement within a unified infrastructure for large-scale usable privacy analysis. The framework transforms heterogeneous consent interactions into reproducible quantitative representations that support the analysis of procedural, attentional, cognitive, and adaptive manipulation mechanisms in dynamic consent ecosystems.

#### 3.2.1. Distributed Browser-Based Acquisition Layer

The Distributed Browser-Based Acquisition Layer provides the foundation for scalable measurement of dynamic consent ecosystems through asynchronous Chromium-based rendering and distributed browser automation. By reconstructing dynamically rendered consent interfaces, iframe-dependent CMP components, and runtime interaction states that are frequently inaccessible to static crawlers, the acquisition layer enables synchronized collection of consent-related artifacts across heterogeneous web environments. Standardized execution configurations and isolated browser sessions ensure scalability, reproducibility, and execution consistency.

The acquisition layer produces synchronized analytical artifacts, including rendered interface structures, visual evidence, network activity, browser storage states, and runtime metadata. These artifacts provide the empirical foundation for subsequent interaction workflow reconstruction, evidence synchronization, consent-state verification, and ACL-DP operationalization within the computational measurement pipeline. The distributed rendering workflow is summarized in Algorithm 1.

**Figure 2:**
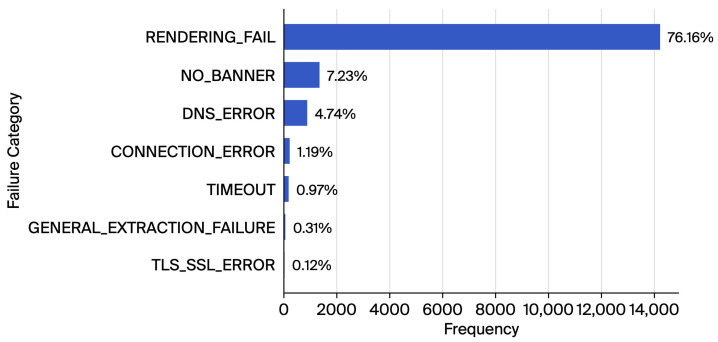
Failure taxonomy distribution in the distributed browser-based acquisition layer.

A principal contribution of the Distributed Browser-Based Acquisition Layer is the establishment of deterministic execution conditions that enable reproducible computational measurement across heterogeneous web environments. Through standardized rendering configurations and a structured failure taxonomy (Figure 2), the framework treats execution reliability as an explicit analytical dimension rather than as uncontrolled experimental noise. This failure-aware acquisition strategy supports systematic evaluation of rendering stability, interaction reconstruction reliability, and infrastructure robustness, thus providing a scalable and reproducible foundation for the analysis of dynamic consent ecosystems.

**Algorithm 1** Asynchronous Distributed Rendering Algorithm
**Require:** Target URL set U**Ensure:** Synchronized rendering artifact set R
  1:Initialize asynchronous worker pool *W*  2:**for all** u∈U **in parallel do**  3:      Launch isolated Chromium execution context  4:      Apply deterministic viewport, locale, and timeout configurations  5:      Navigate to webpage *u*  6:      Wait for rendering stabilization  7:      Extract rendered DOM representation  8:      Capture screenshot evidence  9:      Record network transactions and browser storage events10:      Assign execution status *s*11:      Construct synchronized artifact12:      r←(DOM,Screenshot,Network,Storage,s)13:
**end for**
14:

$R\leftarrow \{r_{1},r_{2},\ldots ,r_{n}\}$

15:**return** R


#### 3.2.2. Human Annotation and Benchmark Construction

Three annotators with complementary evaluation perspectives were selected to conduct the human annotation process, with particular attention to the extent to which cookie-consent interfaces provide users with meaningful consent choices. Each annotator performed the assessments independently following a predefined annotation protocol and a standardized annotation codebook. The annotators were designated as Annotator A, Annotator B, and Annotator C. Subsequently, an analysis of the agreement between the parties was performed to evaluate the reliability and consistency of the annotation process.

The annotation procedure was conducted in three iterative rounds consisting of an initial pilot phase involving 10 domains, followed by two validation phases comprising 50 and 100 domains, respectively. This iterative annotation strategy enabled progressive refinement of the annotation guidelines and assessment criteria. Inter-rater reliability was evaluated using pairwise Cohen’s κ coefficients for annotator pairs (A–B, A–C, and B–C), together with Fleiss’s κ statistics to assess overall agreement among the three annotators. These reliability assessments provided empirical evidence supporting the consistency, reproducibility, and methodological validity of the constructed benchmark dataset. In addition, the benchmark was used to validate three components of the ACL-DP framework: consent banner detection, dark-pattern mechanism classification, and interpretation of overall manipulation severity.

Although agreement coefficients that approach unity are uncommon in many HCI and usability evaluation studies, the exceptionally high agreement observed in this study can be attributed to several methodological characteristics of the ACL-DP framework. First, the annotation process was guided by a highly structured, evidence-based codebook comprising explicit operational definitions and decision criteria. Second, the evaluated constructs primarily captured the characteristics of the consent interface, the interaction mechanisms, and compliance indicators rather than subjective user perceptions or interpretive usability judgments. Third, the annotation protocol incorporated standardized evidence artifacts, including screenshots, consent states, and interaction traces, thus reducing ambiguity and limiting interpretive variability. Consequently, the observed reliability values should be interpreted as evidence of successful operationalization and benchmark standardization rather than as an indication of annotation bias or prior consensus harmonization. Collectively, these findings demonstrate that the ACL-DP framework provides a highly stable, scalable, and reproducible methodological foundation for the systematic evaluation of consent manipulation mechanisms and adaptive cognitive load dark patterns.

#### 3.2.3. Dynamic Interaction Sensing Layer

The Dynamic Interaction Sensing Layer reconstructs consent workflows through runtime interaction monitoring and navigation-state analysis. Unlike static interface inspection, this layer models consent interaction as a sequence of observable states and user actions, including first-layer (L1) banners, second-layer (L2) preference interfaces, disclosure structures, toggle-based controls, and brake navigation paths. This design enables robust reconstruction of dynamic consent workflows across heterogeneous and asynchronously rendered CMP environments.

(1) $$T=s_{0},a_{1},s_{1},\ldots ,a_{n},s_{n},$$

where $s_{i}$ denotes an observable interface state and $a_{i}$ denotes the interaction action that produces a transition between states. The trajectory *T* therefore represents the evolution of a consent workflow as alternating states and actions.

Using the dynamic workflow reconstruction procedure summarized in Algorithm 2, the framework detects actionable consent controls, executes interaction actions, waits for asynchronous rendering stabilization, and records the resulting state transitions.

**Algorithm 2** Dynamic Workflow Reconstruction Algorithm
**Require:** Initial interface state $s_{0}$**Ensure:** Reconstructed interaction trajectory $T=s_{0},a_{1},s_{1},\ldots ,a_{n},s_{n}$
  1:Capture initial rendered interface state $s_{0}$  2:Detect actionable consent elements using selector, XPath, and keyword matching  3:Initialize trajectory storage $T\leftarrow s_{0}$  4:**for all** interaction actions $a_{i}$ **do**  5:      Execute action $a_{i}$  6:      Wait for asynchronous rendering stabilization  7:      Detect resulting state transition  8:      Capture updated interface state $s_{i}$  9:      Record interaction metadata10:      Append transition $\left(s_{i-1},a_{i},s_{i}\right)$ to *T*11:
**end for**
12:**return** *T*


Procedural interaction burden is quantified using cumulative interaction complexity:

(2) $$IC=\sum_{i=1}^{n}w\left(a_{i}\right),$$

where IC denotes the cumulative interaction cost, $a_{i}$ represents the *i*-th action, and $w(a_{i})$ denotes the procedural weight assigned to that action. This measure captures the navigational and interaction effort imposed during the consent process.

Navigation Depth (ND) is operationalized as the deepest consent layer reached during workflow reconstruction:

(3) $$ND=\max_{i}\left(L_{i}\right),$$

where $L_{i}$ denotes the interaction layer observed at workflow step *i*. Unlike click-count measures, ND captures the deepest observable interaction state reached during consent navigation. The interaction layers were encoded as follows:

- L=0: no observable consent interaction transition;
- L=1: interaction confined to the first-layer consent interface;
- L=2: interaction reaching a second-layer preference interface.

To quantify procedural asymmetry between acceptance- and rejection-oriented workflows, click asymmetry is defined as

(4) $$CA=\frac{Clicks_{\mathrm{Reject}}}{Clicks_{\mathrm{Accept}}},$$

where $Clicks_{\mathrm{Reject}}$ and $Clicks_{\mathrm{Accept}}$ denote the number of interactions required to reject and accept consent, respectively. Positive values indicate a greater procedural burden for rejection. Together, IC, ND, and CA support the computational assessment of the interaction burden, procedural asymmetry, and the complexity of the consent process.

#### 3.2.4. Multi-Layer Synchronization Layer

The Multi-Layer Synchronization Layer integrates interface observations, reconstructed interaction trajectories, textual evidence, and consent-state signals into a unified analytical representation. This synchronization enables the framework to examine how interface design, user interaction pathways, disclosure content, and backend consent behavior jointly shape dynamic consent ecosystems.

The synchronized representation is defined as

(5) $$D={\left\{\left(U_{i},I_{i},T_{i},C_{i}\right)\right\}}_{i=1}^{n},$$

where $U_{i}$ denotes interface-layer observations, $I_{i}$ represents interaction trajectories, $T_{i}$ denotes textual and semantic features, and $C_{i}$ represents consent-state and backend-aligned evidence.

A central function of this layer is the operationalization of attentional prioritization through a visual salience model:

(6) $$VW=S+C_{v}+P+F,$$

where VW denotes visual weight, *S* represents relative button size, $C_{v}$ denotes contrast visibility, *P* represents positional salience, and *F* denotes font emphasis.

The Attention Engineering prominence ratio is computed as

(7) $$AE_{\mathrm{Prominence}}=\frac{VW_{\mathrm{Accept}}}{VW_{\mathrm{Reject}}},$$

where $VW_{\mathrm{Accept}}$ and $VW_{\mathrm{Reject}}$ denote the visual weights assigned to acceptance- and rejection-oriented controls, respectively. Values above one indicate a greater visual prominence of acceptance-oriented controls.

The synchronization procedure, summarized in Algorithm 3, combines attentional, procedural, textual, and backend evidence into multidimensional feature vectors for subsequent ACL-DP scoring and statistical analysis.

**Algorithm 3** Visual Salience and Multi-Layer Synchronization Algorithm
**Require:** UI-layer representations *U*, interaction trajectories *I*, textual representations *T*, consent-state artifacts *C***Ensure:** Synchronized multidimensional analytical representation *D*
  1:Compute size salience score *S*  2:Compute contrast visibility score $C_{v}$  3:Compute positional salience score *P*  4:Compute font-emphasis score *F*  5:Compute visual weight $VW=S+C_{v}+P+F$  6:Compute $AE_{\mathrm{Prominence}}=VW_{\mathrm{Accept}}/VW_{\mathrm{Reject}}$  7:Extract interaction features from *I*  8:Extract textual complexity features from *T*  9:Extract consent-state synchronization features from *C*10:**for** i=1 to *n* **do**11:      Construct synchronized representation $D_{i}=\left(U_{i},I_{i},T_{i},C_{i}\right)$12:
**end for**
13:Store synchronized analytical feature vectors14:**return** $D=D_{1},D_{2},\ldots ,D_{n}$


To reduce dependence on arbitrary scoring assumptions, the ACL-DP weighting and thresholding procedures were explicitly defined and evaluated by sensitivity analysis. Indicators were assigned to one of four mechanism families: Effort Engineering (EE), Attention Engineering (AE), Cognitive Load Amplification (CL), and Algorithmic Adaptivity (AA). Binary indicators were encoded as 0 or 1, ordinal indicators were normalized to [0,1], and continuous indicators were scaled using bounded min–max normalization. The Mechanism-family scores were then calculated by aggregating normalized indicators within each family, and the composite ACL-DP score was obtained by weighted aggregation of EE, AE, CL, and AA.

Equal weighting was adopted as the baseline strategy because no validated theoretical or empirical evidence currently establishes a stable hierarchy among the four mechanism families. This provides a transparent and reproducible baseline while reducing the risk of overfitting the scoring model to the observed dataset.

Robustness was assessed using alternative theory-informed weighting schemes, randomized weight perturbation, exclusion of the AA mechanism family, and threshold-shift analyses for Low, Medium, and High manipulation-intensity categories. Rank stability was evaluated using Spearman’s ρ, while category stability was assessed using percentage agreement and Cohen’s κ. These analyzes indicated that the principal ACL-DP findings remained stable across alternative weinalysesghting and threshold configurations.

#### 3.2.5. Consent-State Verification Layer

The Consent-State Verification Layer evaluates whether visible consent choices are consistently propagated to browser storage, network communication, consent signals, runtime APIs, and backend persistence mechanisms. This layer extends interface-level analysis by identifying discrepancies between observable frontend consent actions and backend consent-state behavior.

Backend consent propagation is represented as a temporal sequence of observable consent states:

(8) $$CS_{t}={c_{i}}_{i=0}^{n},\;c_{i}\in C,$$

where $CS_{t}$ denotes the temporal consent-state trajectory and $c_{i}$ represents the observable backend consent state at interaction step *i*.

The backend verification procedure, summarized in Algorithm 4, captures storage artifacts, network synchronization events, consent tokens, vendor identifiers, and tracking requests. It then compares frontend consent actions with corresponding backend consent-state evidence.

Frontend–backend consent mismatch is defined as

(9) CM=I(Frontend≠Backend),

where CM denotes the consent mismatch indicator and I(·) is defined as

(10) $$I\left(\mathrm{Frontend}\neq \mathrm{Backend}\right)=\left\{\begin{array}{cc} 1, & \mathrm{if}\;\mathrm{the}\;\mathrm{observable}\;\mathrm{frontend}\;\mathrm{consent}\;\mathrm{state}\;\mathrm{differs}\;\mathrm{from}\;\mathrm{the}\;\mathrm{backend}\;\mathrm{consent}\;\mathrm{state},\\ 0, & \mathrm{otherwise}. \end{array}\right.$$

A value of CM=1 indicates a divergence between the user-visible consent condition and the synchronization behavior of the backend, while CM=0 indicates consistency.

**Algorithm 4** Backend Consent Verification Algorithm
**Require:** Observable frontend consent state *F*, observable backend consent state *B***Ensure:** Consent mismatch indicator CM
  1:Capture browser storage artifacts  2:Capture network synchronization events  3:Capture consent tokens, vendor identifiers, and tracking requests  4:Reconstruct backend consent-state trajectory $CS_{t}=c_{0},c_{1},\ldots ,c_{n}$  5:Compare frontend and backend consent conditions  6:**if** F≠B **then**  7:      CM←1  8:
**else**
  9:      CM←010:
**end if**
11:Store backend-state transitions and synchronization events12:**return** CM


Consent-mismatch analysis required complete aligned evidence linking frontend actions with backend consent-state representations. Therefore, an environment was included only when four types of evidence were available: observable frontend consent actions, browser storage updates, network transmission behavior, and persistent backend consent-state evidence. Although backend verification was performed across 9998 environments, only 161 environments satisfied all evidentiary requirements for the direct consent-mismatch assessment. Consequently, consent-mismatch percentages were calculated relative to the measurable synchronization subset (n=161), not the full backend verification population or the overall acquisition population.

Collectively, the interaction sensing, synchronization, and consent-state verification layers transform dynamic consent workflows into reproducible quantitative measurements for analyzing procedural burden, attentional prioritization, frontend–backend consistency, and observed adaptive consent behavior.

#### 3.2.6. Implementation Details and Quality-Control Procedures

The computational pipeline combined structural, textual, and visual evidence to detect consent interfaces and actionable consent controls. Candidate consent elements were identified using CSS selectors, XPath expressions, ARIA roles, button attributes, modal-dialog structures, and DOM text matching. The detection procedure searched for common consent-related terms associated with acceptance, rejection, management, preferences, cookie settings, privacy options, and vendor choices.

To reduce language bias, multilingual keyword dictionaries were used for common consent actions, including acceptance, rejection, continuation, management, and preference configuration. Labels were normalized by lowercasing, removing punctuation, trimming whitespace, and matching against language-specific synonym sets. When exact matching failed, partial string matching and semantic proximity to consent-related terms were used conservatively.

iframe handling was performed through recursive inspection of accessible frames. The crawler first inspected the main DOM and then traversed same-origin and accessible cross-frame contexts to identify embedded CMP interfaces. When iframe content was inaccessible due to browser security restrictions, cross-origin limitations, or script isolation, the case was flagged as iframe-dependent and treated separately in the failure and uncertainty analysis.

Anti-bot and acquisition failures were classified according to observable execution outcomes, including timeout, CAPTCHA or challenge page, blocked navigation, script failure, empty DOM, rendering failure, and inaccessible consent interface. These cases were not treated as negative consent detections unless sufficient evidence indicated that there was no consent banner.

The false-positive control was performed through three checks. First, detected consent elements were required to appear within a consent-relevant DOM context, such as a cookie banner, privacy modal, CMP container, or preference dialog. Second, screenshot evidence and DOM text were compared to verify that detected controls corresponded to consent-related interface elements. Third, a human-annotated benchmark was used to evaluate whether computational detections aligned with expert judgments for banner presence, rejection availability, and dark-pattern classification.

#### 3.2.7. ACL-DP Computational Operationalization Layer

The ACL-DP Computational Operationalization Layer transforms synchronized multidimensional interaction representations into quantifiable computational constructs to enable scalable behavioral analysis and consent ecosystem evaluation. Specifically, the framework operationalizes ACL-DP through four mechanism families: EE, AE, CL, and AA. These mechanism families capture complementary dimensions of manipulative interaction design, including procedural burden, attentional steering, cognitive processing demands, and adaptive behavioral modulation, respectively. The ACL-DP framework is a rule-based, integrated computational measurement system designed to acquire, reconstruct, synchronize, and operationalize observable consent behaviors.

Algorithmic Adaptivity (AA) was defined as the extent to which a cookie-consent interface or consent-state mechanism exhibited observable runtime, context-dependent, session-sensitive, or backend-mediated variation, unlike EE, AE, and CL, which primarily capture observable interface burden and presentation characteristics. AA was operationalized using observable indicators extracted from the dynamic workflow reconstruction and backend verification stages. These indicators included (1) runtime banner variation across page reloads or sessions; (2) session-state dependency, where the interface changed after previous interaction or stored consent state; (3) geolocation- or locale-sensitive consent configuration; (4) dynamic L2 preference reconstruction; (5) consent-state persistence variation across browser storage mechanisms; (6) CMP/API response variation; and (7) frontend–backend synchronization anomalies.

The Algorithmic Adaptivity (AA) mechanism was operationalized using observable indicators of runtime behavioral variation, including session-dependent behavior, interface reconstruction, context-sensitive consent presentation, and backend-mediated consent-state changes. Each indicator was encoded as a normalized binary or ordinal variable and aggregated into a composite AA subscore according to Equation (11). To avoid overestimating adaptive behavior, missing indicators were treated conservatively and were not interpreted as evidence of adaptivity unless supported by observable runtime interaction patterns or backend verification evidence.

(11) $$AA=\frac{1}{m}\sum_{j=1}^{m}a_{j},$$

where $a_{j}$ denotes the normalized value of the characteristic of *j*-th Algorithmic Adaptivity (AA), and *m* denotes the number of observable AA indicators available within the consent environment. Missing AA indicators were treated conservatively and were not interpreted as evidence of adaptivity unless there was observable runtime behavior or backend evidence.

The computational operationalization of the ACL-DP framework transformed synchronized behavioral observations into standardized mechanism-level representations suitable for quantitative analysis. The operationalization procedure involved normalization of heterogeneous interaction variables, computation of the four ACL-DP mechanism scores (EE, AE, CL, and AA), and aggregation into a composite ACL-DP manipulation score. The complete computational procedure is formally specified in Algorithm 5.

Because composite indicators are sensitive to weighting assumptions, the ACL-DP framework adopted an explicitly defined and reproducible weighting strategy. Equal weighting was selected as the primary aggregation approach for three reasons. First, no validated theoretical framework currently provides quantitative estimates of the relative influence of EE, AE, CL, and AA on consent decision-making behavior. Second, empirical estimation of mechanism-specific weights would require independent ground-truth behavioral outcomes that remain unavailable in contemporary consent ecosystem datasets. Third, the literature on composite indicators recommends equal weighting as a transparent, reproducible, and methodologically conservative baseline when theoretical or empirical evidence for differential weighting is lacking.

To evaluate the robustness of the weighting strategy, sensitivity analyzes were performed using theory-informed weighting configurations and randomized weight perturbation experiments. The resulting ACL-DP classifications and mechanism-level scores remained stable across plausible weighting schemes, supporting the robustness of the proposed computational operationalization framework.

The reproducibility and robustness of the ACL-DP framework were evaluated using three complementary dimensions. First, computational reproducibility was assessed through three independent executions of the complete ACL-DP pipeline under identical experimental conditions, including browser configuration, network environment, geographic localization, and execution parameters. Agreement was evaluated across domain acquisition outcomes, dynamic workflow reconstruction, ACL-DP mechanism classification, and consent-state verification results. Second, human annotation reproducibility was evaluated using independent assessments conducted by three annotators, with agreement quantified using pairwise Cohen’s κ and Fleiss’s κ statistics. Third, methodological robustness was assessed by failure-pattern consistency analysis, execution variance estimation, and benchmark stability assessment across repeated executions. Together, these analyses enabled the evaluation of both computational reproducibility and operational stability under repeated experimental conditions.

**Algorithm 5** ACL-DP Composite Mechanism Scoring Algorithm
**Require:** Synchronized multidimensional representation $D={\left\{U_{i},I_{i},T_{i},C_{i}\right\}}_{i=1}^{n}$**Ensure:** Composite ACL-DP score $ACLDP_{\mathrm{TOTAL}}$
1:Normalize analytical variables$$X=\{x_{1},x_{2},\ldots ,x_{n}\}$$2:**for all** mechanisms $M_{k}\in \left\{EE,AE,CL,AA\right\}$ **do**3:      Compute mechanism score$$M_{k}=\sum_{i=1}^{n}w_{i}\;z\left(x_{i}\right)$$4:
**end for**
5:Compute composite ACL-DP score$$ACLDP_{\mathrm{TOTAL}}=w_{1}EE+w_{2}AE+w_{3}CL+w_{4}AA$$6:Store mechanism-level representations$$\left\{EE,AE,CL,AA,ACLDP_{\mathrm{TOTAL}}\right\}$$7:**return** $ACLDP_{\mathrm{TOTAL}}$


In general, the ACL-DP Computational Operationalization Layer establishes a unified analytical infrastructure that transforms synchronized interaction behaviors into measurable computational representations. By integrating interaction trajectories, synchronized behavioral features, consent-state verification, and mechanism-level operationalization, the framework enables scalable behavioral privacy analytics, adaptive consent monitoring, and systematic evaluation of dynamic consent ecosystems.

## 4. Results

### 4.1. Large-Scale Web Acquisition Performance

The Distributed Browser-Based Acquisition Layer provided a scalable infrastructure for acquiring dynamic cookie-consent ecosystems through asynchronous Chromium-based rendering and distributed browser automation. This layer enabled synchronized collection of rendered interface states, visual evidence, network activity, browser storage artifacts, and execution metadata across heterogeneous web environments. Standardized browser configurations, deterministic execution parameters, and resumable acquisition workflows ensured reproducible acquisition under controlled experimental conditions, thus establishing the foundation for workflow reconstruction, multi-layer synchronization, and ACL-DP feature extraction (Figure 2).

Acquisition metadata confirmed that all browser sessions were executed under standardized rendering conditions, including fixed viewport dimensions, locale settings, timeout thresholds, and execution environments. Repeated execution experiments demonstrated stable acquisition behavior and consistent reconstruction outcomes across heterogeneous consent ecosystems, supporting the scalability and reproducibility of the proposed computational measurement framework.

The acquisition layer generated synchronized analytical artifacts, including rendered interface structures, screenshots, network logs, storage observations, and execution metadata across 18,665 domains. As shown in Table 1, successful rendering was achieved for 3139 environments (16.82%), while acquisition and rendering failures remained the primary constraints on the observability of the consent ecosystem. Among successfully rendered environments, observable consent banners were identified in 1789 cases (56.99%), and Layer-2 preference systems were detected in 810 environments (25.80%), confirming that successful rendering is a prerequisite for subsequent interaction sensing and workflow reconstruction.

Rendering-related failures, as illustrated in Figure 2, represented the majority of unsuccessful acquisitions and substantially exceeded DNS, connection, and timeout failures, underscoring the challenges associated with dynamic and interaction-dependent consent architectures. Sector-level analyzes also revealed heterogeneous observability patterns, suggesting that rendering performance varies according to implementation characteristics and CMP deployment strategies.

Table 1 summarizes the stage-specific analytical populations generated by the ACL-DP framework, which should not be interpreted as a strictly sequential attrition process. The acquisition stage included 18,665 environments, of which 3139 were successfully rendered and constituted the reference population for consent-banner and Layer-2 analyses. Dynamic workflow reconstruction yielded 2304 interaction records, while backend verification was performed independently for 9998 environments with observable consent-state behavior.

**Figure 3:**
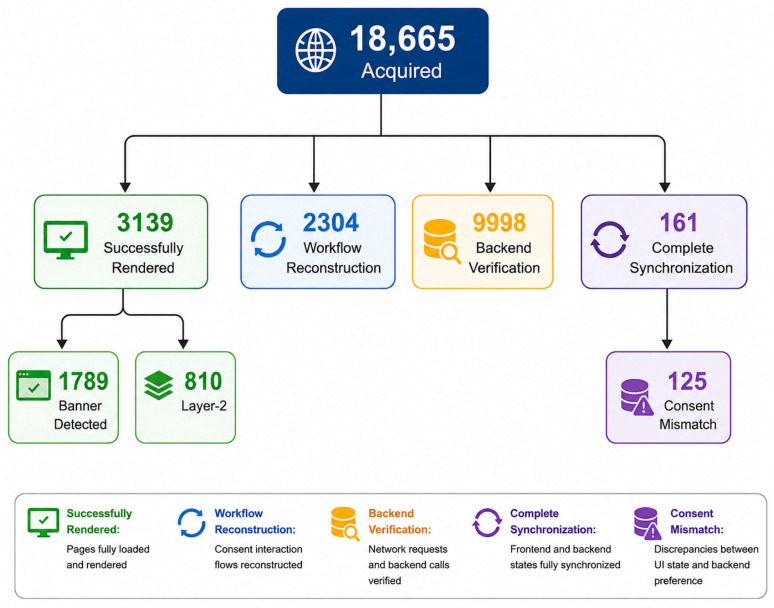
Population Distribution.

Consent mismatch analysis required synchronized observation of frontend interactions, browser storage changes, network activity, and backend consent-state persistence. Only 161 environments satisfied these evidentiary criteria; therefore, consent mismatch estimates are reported relative to this measurable synchronization subset and should not be interpreted as population-wide prevalence estimates Figure 3.

### 4.2. Human Annotation Benchmark Evaluation

Human annotation reproducibility demonstrated exceptionally high levels of agreement across the principal dimensions of the ACL-DP framework. Pairwise Cohen’s κ coefficients ranged from 0.937 to 1.000 across the three annotator pairs (Annotators A–B, A–C, and B–C), indicating almost-perfect agreement. Similarly, multi-rater Fleiss’s κ coefficients ranged from 0.958 to 1.000 across the principal annotation dimensions, demonstrating near-perfect consistency among annotators. The only observed discrepancies occurred within a small number of GDPR indicator assessments, where an annotator classified several interfaces as “Unclear” rather than “Weak”, reflecting semantic interpretation differences rather than substantive conceptual disagreement. Overall, these findings provide strong empirical evidence supporting the reproducibility, reliability, and methodological validity of consent banner detection, ACL-DP mechanism classification, and manipulation severity assessment.

At the benchmark level, the ACL-DP framework achieved a mean Cohen’s κ of 0.989 and a mean Fleiss’s κ of 0.990, indicating a highly consistent interpretation and application of the operational definitions. Failure-pattern consistency was similarly high (FPC = 0.975), demonstrating that the annotators consistently distinguished between the assessable, ambiguous, and evidence-limited consent interfaces. The benchmark stability analysis also revealed a consensus rate of 97.5%, an adjudication burden of only 2.5%, and no instances of severe disagreement, collectively indicating exceptional benchmark stability and reproducibility. The high agreement structure of the benchmark was further reflected in the consistent annotation of Attention Engineering, Cognitive Load, GDPR Indicators, and Overall Risk across all annotation rounds.

### 4.3. Workflow Reconstruction Performance

The Dynamic Interaction Sensing Layer functioned as a behavioral sensing infrastructure that reconstructed consent workflows through runtime interaction monitoring and navigation-state analysis. By transforming heterogeneous consent interfaces into synchronized behavioral trajectories comprising L1 and L2 structures, consent controls, and interaction pathways, the framework allowed computational analysis of procedural burden, navigation asymmetry, and workflow complexity across dynamic consent ecosystems.

The interaction sensing architecture achieved a workflow reconstruction success rate of 99.61%, demonstrating robust reconstruction performance after successful rendering acquisition (Table 2). Only nine workflows failed due to interaction-level execution instability. Furthermore, 79.56% of reconstructed environments contained actionable consent controls suitable for behavioral analysis.

#### 4.3.1. Interaction-State Trajectories

The framework represented reconstructed consent workflows as sequential behavioral trajectories based on Equation (1). Most workflows consisted of an initial state and a synchronized post-interaction state. Descriptive statistics indicate that typical workflows were relatively short, with median values of one action, one navigation layer, and IC=1. However, interaction complexity exhibited substantial right-skewness, with a 95th percentile value of 22 and a maximum of 2466, reflecting highly complex L2 preference structures.

Table 3 summarizes workflow complexity, navigation depth, interaction burden, interface control density, and iframe prevalence. The Navigation Depth metric exhibited a mean of 0.67 and a median of 1.00, indicating that most environments did not involve observable interaction transitions (ND=0) or only interactions in the first-layer (ND=1), consistent with the observed distribution of the reconstructed consent workflows.

#### 4.3.2. L1 and L2 Navigation Structures

Table 4 summarizes the distribution of observable consent controls L1 reconstructed during the workflow analysis. The results revealed pronounced procedural asymmetry, with acceptance-oriented controls substantially more visible than rejection-oriented alternatives. In particular, the “Accept All” controls were approximately 6.47 times more prevalent than the “Reject All” controls, indicating a strong accessibility advantage favoring acceptance pathways.

#### 4.3.3. L2 Preference System Complexity

L2 preference structures were observed in a subset of reconstructed workflows and exhibited substantial variability in procedural and informational complexity. As summarized in Table 5, these environments contained toggle-based controls, vendor-specific disclosures, category-level consent options, and expandable preference sections, reflecting the complexity of advanced consent management interfaces.

Although L2 controls were present in a minority of reconstructed environments, their scale varied considerably across consent ecosystems, indicating substantial heterogeneity in preference-management design. Most workflows exhibited relatively low procedural complexity; however, a small subset contained exceptionally dense preference-management structures characterized by large numbers of toggle controls, vendor disclosures, category-level options, and expandable sections. The most complex observed environments contained thousands of toggles and hundreds of expandable disclosure elements, demonstrating that the procedural burden was concentrated within a limited number of highly complex consent ecosystems rather than being uniformly distributed throughout the dataset.

These findings suggest that the consent burden arises not only from the interaction effort but also from the complexity of configuration spaces, vendor lists, consent categories, and expandable preference architectures. Consequently, L2 preference systems represent an important source of procedural and cognitive burden within contemporary consent ecosystems.

### 4.4. Cross-Layer Synchronization Performance

The Multi-Layer Synchronization Layer integrated UI-layer features, interaction trajectories, textual complexity indicators, and backend consent-state evidence into a unified analytical dataset based on Equation (5). The synchronized dataset contains 18,665 analytical records and 59 variables.

#### 4.4.1. Synchronized Dataset Construction

The synchronized dataset preserved both observable and non-observable execution outcomes, thereby enhancing reproducibility through the retention of complete acquisition and reconstruction contexts. The synchronized dataset contains post-processing status categories that differ from the execution outcomes recorded at the acquisition stage. As summarized in Table 6, the dataset captures successful acquisitions, rendering failures, non-banner environments, network interruptions, timeout conditions, and interaction reconstruction failures encountered during ecosystem-wide consent analysis.

#### 4.4.2. Visual Salience and Attentional Prioritization

Table 7 summarizes AE metrics derived from synchronized interface representations. The results show a strong visibility imbalance: accept controls appeared approximately 6.03 times more often than reject controls. Among records where both visual weights could be compared, 74.59% favored acceptance-oriented controls.

#### 4.4.3. Procedural Asymmetry Synchronization

The click asymmetry was calculated on the basis of Equation (4). Effort Engineering (EE) indicator descriptive statistics were extracted from synchronized interaction trajectories. Table 8 summarizes navigation depth, interaction complexity, click asymmetry, and aggregated EE score used to operationalize procedural burden and navigational friction within consent interaction environments. Positive click asymmetry appeared in 1050 records (5.63%), indicating cases where rejection required more interaction effort than acceptance. Although the dataset-wide mean CA was close to zero, this is because the full synchronized file includes many non-interactive records. The non-zero subset confirms the presence of measurable procedural asymmetry.

#### 4.4.4. Textual Complexity Synchronization

The textual burden was concentrated in a subset of consent ecosystems. Although the median word count was zero due to many non-observable cases, the maximum disclosure length reached 2446 words, and the 95th percentile reached approximately 1190 words, indicating that high-burden disclosure environments were present. Table 9 summarizes Cognitive Load Amplification indicators extracted from synchronized textual representations.

#### 4.4.5. Toggle–Vendor Complexity Synchronization

The results show that the toggle and vendor complexity were not universal but appeared in concentrated high-complexity cases. This supports the interpretation that CL emerges in specific ecosystems through dense preference structures, vendor lists, category fragmentation, and disclosure expansion. Table 10 shows the descriptive statistics of the consent-state complexity indicators extracted from the L2 preference systems. The metrics reported quantify the prevalence of toggle-based controls, vendor-specific disclosures, category-level consent options, and disclosure complexity characteristics used to operationalize consent-state complexity within synchronized consent interaction environments.

#### 4.4.6. Consent-State Synchronization

The synchronization layer successfully integrated backend-state evidence into the unified feature space. Although backend observability was not present in all records, the non-zero subset demonstrated measurable storage and network synchronization activity. The consent-state verification results and consent-state complexity metrics were extracted during the backend synchronization analysis. Table 11. Panel A summarizes the prevalence of observable consent states, third-party tracking activity, storage artifacts, and network-level tracking evidence. Panel B reports descriptive statistics for the storage-state activity, network synchronization events, and the aggregated consent-state complexity score used within the Consent-State Verification Layer.

#### 4.4.7. Composite Synchronization Scores

The composite results show that Effort Engineering was the family of observable mechanisms that was observed more consistently, while Attention Engineering, Cognitive Load, and Consent-state complex appeared more selectively in subsets of synchronized environments. The descriptive statistics of the ACL-DP composite mechanism scores are described in Table 12. The distribution of EE, AE, CL, Consent-State Complexity (CSC), and the aggregated ACL-DP Total Score used to quantify the manipulation intensity across synchronized consent interaction environments.

### 4.5. Consent-State Verification Performance

#### 4.5.1. Backend Verification Execution Outcomes

The backend verification layer processed 9998 records, of which 1951 were successfully verified, corresponding to 19.51% of the verification corpus. Table 13 summarizes the results of the backend consent-mismatch detection analysis, including successful verification processes, Stage 5 processing failures, navigation-related failures, and timeout conditions encountered during consent-state propagation of the consent-state and the backend verification.

The relatively low success rate reflects the greater technical difficulty of backend-state verification compared with frontend interface detection. This layer required the simultaneous capture of interaction states, cookies, storage artifacts, network propagation behavior, and backend consent indicators within a synchronized runtime environment.

#### 4.5.2. Consent Mismatch Analysis

The results of the detection of consent mismatch obtained during the verification of the consent state in the backend are summarized in Table 14. The evaluation of mismatch required complete aligned evidence linking observable frontend consent states with the corresponding backend propagation behavior. Although backend monitoring was conducted in 9998 environments, only 161 records contained sufficient frontend–backend aligned evidence to support a reliable mismatch assessment. Frontend–backend synchronization analysis required the simultaneous observation of user interface interactions, browser storage updates, network transmissions, and consent-state persistence mechanisms. Due to implementation heterogeneity across consent management platforms, encrypted storage architectures, asynchronous event propagation, and server-side consent processing, complete synchronization reconstruction was feasible only for a subset of backend-verified environments. Consequently, consent mismatch analysis was restricted to environments with fully observable synchronization traces, and the resulting synchronization subset should be interpreted as an observationally reconstructable population rather than a random sample of all backend-verified consent environments. Among the 9998 environments verified by the backend, 161 contained sufficient synchronized frontend and backend evidence for direct consent-mismatch assessment. Within this measurable synchronization subset, 125 cases exhibited consent mismatch, corresponding to 77.64% (95% CI: 70.47–83.47). This percentage refers only to the evaluable synchronization subset and not to all backend-verified or acquired environments, rather than population prevalence measures.

#### 4.5.3. Data Quality and Pipeline Reliability

The implementation-level quality-control procedures reduced the risk of treating unrelated pop-ups, newsletter prompts, advertisements, or generic modal dialogs as consent interfaces. Cases involving inaccessible iframes, anti-bot challenges, or incomplete rendering were retained as separate uncertainty categories rather than being counted as confirmed negative observations.

### 4.6. ACL-DP Mechanism Analysis

#### 4.6.1. Mechanism-Level Score Distribution

Distributional characteristics of the ACL-DP mechanism family scores. Table 15 reports the central tendency and upper-tail behavior of EE, AE, CL, AA, and the aggregated ACL-DP score. The normalized ACL-DP score is also reported on a scale of 0 to 100 to facilitate interpretability and comparative assessment of the intensity across synchronized consent interaction environments. Effort Engineering was the most consistently observable ACL-DP mechanism. This indicates that the procedural burden, including click asymmetry, hidden rejection pathways, navigation depth, and interaction friction, represented the dominant mechanism family in the consent ecosystems analyzed.

#### 4.6.2. ACL-DP Intensity Classification

Distribution of ACL-DP intensity categories across the analyzed consent environments (Table 16). Intensity levels were derived from the normalized ACL-DP composite score and represent the general degree of manipulation associated with the EE, AE, CL, and AA mechanisms. The results indicate that the majority of the environments observed consent exhibited low manipulation intensity, but 671 environments reached Moderate or High levels, indicating a smaller subset of ecosystems where multiple manipulation mechanisms co-occurred. AA showed a positive and statistically significant association with the composite ACL-DP score ((ρ = 0.656), adjusted FDR (*p* < 0.001)). However, AA exhibited weaker associations with EE, AE, and CL than those mechanisms exhibited with each other. This pattern suggests that AA captures a partially distinct dimension of consent-interface behavior related to runtime and backend-mediated adaptivity rather than simply duplicating static interface complexity.

#### 4.6.3. Highest-Scoring ACL-DP Ecosystems

Top-ranked consent environments according to the ACL-DP composite score. Table 17 reports the highest-scoring domains together with their corresponding EE, AE, CL, and AA scores. The normalized ACL-DP score (0–100 scale) facilitates a comparative assessment of manipulation intensity across synchronized consent interaction ecosystems. The highest-scoring ecosystems did not show elevation in a single mechanism. Instead, they combined multiple ACL-DP dimensions, especially Effort Engineering and Attention Engineering, with additional cognitive load or adaptivity signals.

#### 4.6.4. Statistical Association Results

Spearman correlation analysis between ACL-DP mechanism-family scores and the composite ACL-DP score. The correlation coefficients were evaluated using the false discovery rate (FDR) correction. All mechanism families exhibited statistically significant positive associations with the overall ACL-DP score, indicating that EE, AE, CL, and AA each contribute substantially to the multidimensional operationalization of manipulation intensity. The results of The Spearman correlation presented in Table 18 showed strong positive associations between the mechanisms family scores and the composite ACL-DP score.

Spearman correlation coefficients among ACL-DP mechanism families after false discovery rate (FDR) correction. The results indicate strong positive associations among EE, AE, and CL, while AA exhibits moderate but statistically significant associations with the remaining mechanism families. These findings suggest that multiple manipulation mechanisms frequently co-occur within synchronized consent interaction environments. The results, presented in Table 19, indicate that ACL-DP mechanisms frequently co-occur rather than appearing as isolated manipulation features.

## 5. Discussion

### 5.1. Implications of Large-Scale Consent Acquisition

The Distributed Browser-Based Acquisition Layer demonstrated the feasibility of comprehensive consent ecosystem sensing through coordinated browser orchestration and synchronization-based acquisition mechanisms. The predominance of rendering-related failures indicates that contemporary consent environments increasingly depend on asynchronous rendering architectures, dynamically instantiated CMP components, and interaction-dependent interface states that challenge conventional static auditing approaches. These findings highlight the importance of runtime rendering for achieving meaningful observability of consent mechanisms.

From a methodological perspective, the framework contributes to a reproducible acquisition infrastructure by combining deterministic rendering conditions with a structured failure taxonomy (Figure 2). Rather than treating acquisition instability as uncontrolled noise, rendering failures, timeout conditions, and network interruptions were operationalized as measurable execution states, thus improving transparency and reproducibility in computational web measurement studies.

The inclusion of a human-annotated benchmark substantially strengthens the construct validity of the ACL-DP framework. Rather than treating dark-pattern identification as a purely computational problem, the present study operationalizes the ACL-DP assessment as a hybrid human–computational evaluation process grounded in observable interface characteristics and expert judgment. Furthermore, the comparatively lower correlations between AA and the other ACL-DP mechanism families indicate that Algorithmic Adaptivity represents a distinct layer of consent manipulation. Whereas EE, AE, and CL primarily capture visible interface burden, AA captures dynamic and context-dependent behavior that may only become observable through repeated interaction, consent-state verification, and backend synchronization analysis.

From an HCI and usable privacy perspective, results suggest that many consent interfaces remain partially hidden behind rendering dependencies and procedural interaction requirements. Consequently, a meaningful evaluation of consent ecosystems requires synchronized acquisition of rendered interfaces, interaction states, and associated execution artifacts rather than relying on static interface inspection alone.

### 5.2. Implications of Dynamic Consent Workflow Reconstruction

The Dynamic Interaction Sensing Layer transformed rendered consent interfaces into measurable behavioral trajectories, demonstrating that consent ecosystems operate as procedural interaction environments rather than static interface artifacts. The high workflow reconstruction success rate confirms the robustness of synchronization-sensitive interaction sensing across heterogeneous consent architectures.

The relatively low mean Navigation Depth observed in this study reflects the structural characteristics of contemporary consent interfaces rather than limitations of the workflow reconstruction methodology. Specifically, many environments either provide only first-layer consent controls or do not expose observable interaction pathways beyond the initial consent interface, thereby constraining the achievable navigation depth. Within this structural context, two findings are particularly noteworthy. First, acceptance-oriented pathways were substantially more prominent than rejection-oriented alternatives, indicating persistent procedural asymmetry in contemporary consent workflows. Second, although most workflows exhibited relatively low structural complexity, a subset of environments contained highly complex preference-management interfaces characterized by numerous toggle controls, extensive vendor disclosures, and multiple expandable sections.

From an ACL-DP perspective, hidden rejection pathways, navigation depth, and configuration complexity provide measurable evidence of EE and CL, while the visibility imbalance between acceptance and rejection controls reflects AE mechanisms that may steer users toward lower-effort acceptance decisions.

### 5.3. Cross-Layer Consent Synchronization Analysis

The Multi-Layer Synchronization Layer demonstrates that consent ecosystems cannot be adequately understood through isolated interface analysis. Instead, meaningful evaluation requires the synchronized integration of interface visibility, interaction trajectories, disclosure complexity, and backend consent-state evidence within a unified analytical representation.

The relatively small proportion of environments exhibiting fully reconstructable frontend–backend synchronization traces reflects the technical complexity and heterogeneity of contemporary consent ecosystems rather than the rarity of consent inconsistencies. Modern consent architectures frequently employ asynchronous processing, encrypted storage, server-side state management, and proprietary synchronization protocols that limit direct observational reconstruction.

The observed consent-mismatch rate reflects the prevalence of frontend–backend inconsistencies among environments for which complete aligned evidence was available. Consequently, this finding should not be interpreted as a population-level estimate of consent inconsistency across all acquired or backend-verified websites. Rather, it characterizes the subset of environments in which synchronized observations of user interactions, browser states, network activity, and backend consent persistence could be reliably reconstructed. Nevertheless, the high frequency of consent mismatches within this measurable subset indicates that frontend–backend inconsistencies constitute a substantial empirical phenomenon that merits further investigation.

The synchronized dataset revealed that procedural asymmetry, attentional prioritization, textual burden, and consent-state activity frequently co-occur within the same interaction environments. In particular, acceptance-oriented controls were more prominent than rejection-oriented alternatives, while disclosure complexity and preference-management burden were concentrated in a subset of highly complex ecosystems.

Collectively, these findings support the central premise of RQ1: privacy interaction behavior emerges through dependencies among multiple analytical layers, rather than through any single interface characteristic. The synchronization layer therefore provides the computational foundation necessary for multidimensional behavioral modeling and mechanism-aware consent analysis.

### 5.4. Implications of Consent-State Verification

The Consent-State Verification Layer extends conventional consent auditing beyond visible interface inspection by incorporating backend propagation evidence into the analytical process. The results demonstrate that observable consent interactions frequently trigger measurable changes in browser cookies, storage artifacts, tracking requests, and vendor synchronization behavior. A key finding of the analysis is the high prevalence of consent mismatch among environments for which complete frontend–backend aligned evidence was available. Of the 9998 environments subjected to backend verification, only 161 satisfied the evidentiary requirements necessary for direct consent-mismatch assessment, including synchronized observations of frontend interactions, browser storage states, network transmissions, and backend consent persistence. Within this measurable synchronization subset, 125 environments (77.64%) exhibited discrepancies between observable frontend consent conditions and corresponding backend propagation behavior. Consequently, this result should be interpreted as a high mismatch rate among environments with reconstructable aligned evidence, rather than as a population-level estimate of consent inconsistency across the broader web ecosystem. These findings suggest that visible consent interfaces do not always accurately reflect the underlying consent-state behavior. From a privacy engineering perspective, they underscore the importance of integrating frontend interaction sensing with backend verification mechanisms when evaluating consent compliance, transparency, and the behavioral integrity of contemporary consent ecosystems.

### 5.5. Implications of ACL-DP Mechanism Analysis

Sensitivity analysis demonstrated that the main ACL-DP findings remained stable across alternative weighting configurations. Spearman rank correlations between the equal-weighting approach and theory-informed weighting schemes exceeded 0.90, indicating a strong preservation of the relative ACL-DP rankings. Similarly, perturbation analyzes involving ±20% variations in mechanism weights produced only negligible changes in the primary statistical associations and empirical conclusions. These findings indicate that the proposed ACL-DP framework is robust to plausible variations in the weighting strategy.

The ACL-DP Computational Operationalization Layer constitutes the primary analytical contribution of the proposed framework by transforming synchronized interaction, textual, attentional, and consent-state features into measurable mechanism-level indicators. Unlike conventional dark-pattern taxonomies, which primarily rely on descriptive classifications, the ACL-DP framework operationalizes manipulative design strategies as quantitative constructs suitable for computational analysis, statistical inference, and behavioral modeling (Table 20).

Among the four mechanism families, Effort Engineering emerged as the dominant manipulation dimension, indicating that procedural burden, hidden rejection pathways, increased navigation depth, and interaction friction represent the most prevalent manipulative strategies within contemporary consent ecosystems. Although Attention Engineering, Cognitive Load Amplification, and Algorithmic Adaptivity exhibited lower frequencies, they remained substantial contributors to the overall manipulation intensity captured by the ACL-DP framework. The observed associations among the ACL-DP mechanism families further suggest that manipulative strategies rarely occur in isolation. Rather, high-intensity consent environments typically exhibit concurrent procedural, attentional, cognitive, and adaptive manipulation characteristics. These findings support the central theoretical premise of the ACL-DP framework, namely that manipulative behavior emerges through the interaction of multiple synchronized mechanisms rather than through isolated interface attributes.

The ACL-DP benchmark evaluation demonstrated substantial improvements in annotation reproducibility following iterative refinement of the operational definitions and evidence-based codebook. Quantitative reproducibility was further supported by exceptionally high inter-rater agreement (mean Cohen’s κ = 0.989; mean Fleiss’s κ = 0.990), a high failure-pattern consistency (FPC = 0.975), a benchmark consensus rate of 97.5%, a minimal adjudication burden of 2.5%, and the absence of severe disagreements. These findings provide direct empirical evidence of the scalability, reproducibility, and robustness of the ACL-DP framework and address RQ4 by demonstrating that the proposed methodology yields highly stable, reliable, and reproducible benchmark annotations across multiple annotators and annotation rounds.

The results of the sensitivity analysis are consistent with the underlying correlation structure of the ACL-DP mechanism families. Strong positive associations among Effort Engineering, Attention Engineering, Cognitive Load Amplification, and the composite ACL-DP score indicate that these mechanisms capture complementary dimensions of a broader latent manipulation construct. Consequently, moderate variations in the weighting configuration produce only limited changes in the resulting composite scores and ranking structures. Furthermore, the moderate correlations observed for Algorithmic Adaptivity suggest that adaptive manipulation mechanisms provide additional explanatory information beyond procedural, attentional, and cognitive dimensions. Collectively, these findings provide convergent empirical evidence supporting the validity, robustness, and weighting stability of the proposed ACL-DP computational operationalization framework. By operationalizing observable consent behaviors into reproducible computational measures, the ACL-DP framework enables researchers, regulators, auditors, and industry practitioners to conduct scalable privacy auditing, benchmark manipulative consent practices, support regulatory enforcement, and inform the design of trustworthy and user-centric consent ecosystems.

### 5.6. Limitations

Despite its scalability, reproducibility, and analytical robustness, the proposed framework has several limitations. First, ACL-DP indicators are derived from computational sensing and therefore capture observable interaction conditions rather than direct measures of user cognition, trust, privacy perception, or decision quality. Second, acquisition and workflow reconstruction remain dependent on rendering accessibility, anti-automation resistance, and website-specific implementation characteristics, which may affect observability in some consent environments. A complete frontend–backend synchronization analysis was achievable only for a subset of backend-verified environments due to observational constraints associated with proprietary consent architectures, encrypted storage, and asynchronous state propagation. Consequently, consent mismatch estimates should be interpreted as representative of observationally reconstructable environments rather than the broader population of web consent interfaces.

Third, the evaluation was conducted under a deterministic desktop configuration to ensure reproducibility; consequently, interaction behavior may differ across devices, browsers, accessibility settings, geographic regions, and language environments. Fourth, ACL-DP operationalization is based on predefined indicators and weighting strategies, and alternative machine-learning or adaptive feature-selection approaches may provide additional insights. Furthermore, the inclusion of repeated execution experiments and inter-rater reliability analysis strengthens the empirical validity of RQ4. Unlike previous large-scale cookie consent studies that primarily report acquisition success rates, the present study demonstrates both computational reproducibility and observational reproducibility through repeated execution and independent human validation. The ACL-DP framework currently employs equal-weight aggregation because empirically validated feature importance estimates remain unavailable. Although sensitivity analyses demonstrated robust findings across alternative weighting schemes, future work may investigate expert-derived weighting, empirical weighting, and machine-learning-based weighting strategies.

Although the human benchmark demonstrated moderate to substantial agreement across several dimensions, some annotation categories exhibited lower agreement levels, reflecting the inherent subjectivity associated with dark-pattern interpretation and cognitive manipulation. Furthermore, AA should be interpreted with caution because adaptive consent behavior is more difficult to observe than static interface properties. Some adaptive mechanisms may occur through proprietary CMP logic, encrypted storage, server-side processing, or region-specific configuration that cannot be fully reconstructed through browser-side observation alone. Consequently, the AA score reflects observable evidence of runtime or backend-mediated adaptivity rather than definitive proof of intentional algorithmic personalization.

Despite these quality-control procedures, some consent interfaces may remain undetected due to cross-origin iframe restrictions, highly customized CMP implementations, dynamic rendering delays, anti-bot systems, or unsupported languages. Therefore, detection results should be interpreted as observable consent-interface evidence under the specified execution configuration.

Finally, backend verification was limited to browser-observable artifacts, while proprietary server-side processing and cross-device tracking mechanisms remained outside the scope of observation. Future user-centered and cross-platform validation studies would further strengthen external validity and generalizability.

## 6. Conclusions and Future Work

This study introduced a Computational Sensing Framework for Automated Reconstruction and Analysis of Dynamic Consent Interaction Ecosystems. The proposed five-layer architecture integrates distributed acquisition, dynamic interaction sensing, multi-layer synchronization, consent-state verification, and computational operationalization of ACL-DP within a unified analytical infrastructure. The framework successfully reconstructed and analyzed consent interactions across 18,665 environments, generated synchronized multidimensional representations, verified the propagation behavior of backend consent, and operationalized manipulation mechanisms through measurable computational indicators. By incorporating Algorithmic Adaptivity, the ACL-DP framework extends beyond static interface analysis and captures dynamic, session-sensitive, and backend-mediated consent behavior that may influence users’ ability to exercise meaningful privacy choice.

The combination of computational analysis and human benchmark validation provides empirical evidence supporting the reliability, reproducibility, and construct validity of the proposed ACL-DP framework. The results demonstrate that procedural burden, attentional asymmetry, CL, and consent-state behaviors frequently co-occur within dynamic consent ecosystems. Effort Engineering emerged as the predominant ACL-DP mechanism family, while consent-mismatch analysis revealed substantial divergence between observable frontend consent interactions and backend consent-state propagation. These findings suggest that consent ecosystems should be conceptualized as multidimensional behavioral environments characterized by the interaction of procedural, attentional, cognitive, and adaptive mechanisms, rather than as isolated interface components or static compliance artifacts.

Although consent-mismatch analysis was restricted to environments for which complete frontend–backend synchronization could be reliably reconstructed, the high frequency of observed mismatches within this measurable subset highlights an important empirical phenomenon that warrants further investigation. Furthermore, the robustness of the ACL-DP findings in multiple weighting configurations indicates that the main analytical conclusions are not artifacts of a particular aggregation strategy, providing additional support for the stability and validity of the proposed computational operationalization framework.

The study contributes to Human–Computer Interaction, usable privacy, and privacy engineering by providing a scalable and reproducible infrastructure for comprehensive privacy interaction analysis, compliance assessment, and consent monitoring. More broadly, it establishes a computational foundation for mechanism-aware evaluation of consent environments and adaptive cognitive load dark patterns.

Future work incorporating advanced browser instrumentation, platform collaboration, and longitudinal measurement approaches may improve the coverage of synchronization reconstruction. Additionally, future work should focus on user-centered validation of ACL-DP indicators, AI-assisted discovery of emerging manipulation mechanisms, longitudinal monitoring of consent ecosystems, and cross-platform, multilingual evaluation. Additional research may also explore explainable privacy-monitoring systems and adaptive compliance assessment frameworks capable of supporting real-time analysis of dynamic consent environments.

## Figures and Tables

**Table 1:** Analytical populations and reference denominators across the ACL-DP framework pipeline.

Framework Stage	Frequency	Percentage (%)	Reference Population
Acquired environments	18,665	100.00	All acquired
Successfully rendered environments	3139	16.82	Acquired environments
No observable consent banner	1349	7.23	Acquired environments
Acquisition failures	14,177	75.95	Acquired environments
Observable consent banners	1789	56.99	Successfully rendered environments
Layer-2 preference systems	810	25.80	Successfully rendered environments
Dynamic workflow reconstruction	2304	12.34	Acquired environments
Backend verification	9998	53.57	Acquired environments
Measurable consent mismatch	161	1.61	Backend verification records
Consent mismatch detected	125	77.64	Measurable synchronization cases

**Table 2:** Dynamic workflow reconstruction outcomes.

Outcome	Frequency	Percentage (%)
Successfully reconstructed workflows	2295	99.61
Failed workflows	9	0.39
Actionable consent controls detected	1827	79.56

**Table 3:** Descriptive statistics of interaction-state trajectory characteristics.

Variable	Mean	Median	Min	Max
Number of States	1.67	2.00	0	2
Number of Actions	0.80	1.00	0	2
Navigation Depth (ND)	0.67	1.00	0	1
Interaction Complexity (IC)	5.25	1.00	0	2466
Visible L1 Control Count	90.24	66.00	0	1956
Initial iframe Count	3.01	1.00	0	55

**Table 4:** Distribution of observable Layer-1 (L1) consent controls across reconstructed consent environments.

L1 Consent Control	Frequency	Percentage (%)
Accept All control present	1656	71.88
Reject All control present	256	11.11
Manage Preferences control present	835	36.24
Hidden rejection pathway detected	687	29.82

**Table 5:** L2 preference system complexity indicators.

L2 Complexity Indicator	Count	Percentage (%)	Mean	Median	Max
	Percentage (%)	Mean	Median	Max	
Toggle Count	467	20.27	4.45	0	2465
Vendor Keyword Hits	485	21.05	1.74	0	120
Category Keyword Hits	530	23.00	4.04	0	371
Expandable Section Count	596	25.87	6.13	0	706

**Table 6:** Execution status distribution in the synchronized dataset.

Status	Frequency	Percentage (%)
RENDER_FAIL	12,257	65.67
SUCCESS	2766	14.82
NO_BANNER	1676	8.98
NETWORK_ERROR	1556	8.34
TIMEOUT	310	1.66
INTERACTION_FAIL	100	0.54

**Table 7:** Attention Engineering (AE) visual prominence metrics.

Metric	Value
Accept Control Present	2587/18,665=13.86%
Reject Control Present	429/18,665=2.30%
Accept Visually Prioritized	320/18,665=1.71%
Valid AE Prominence Ratio Records	429
Mean AE Prominence Ratio	1.05
Median AE Prominence Ratio	1.04
Maximum AE Prominence Ratio	1.61
AE Ratio >1 Among Valid Records	320/429=74.59%

**Table 8:** Effort Engineering metrics derived from synchronized interaction representations.

Metric	Mean	Median	Max	Non-Zero (%)
Navigation Depth (ND)	0.31	0.00	2	24.33
Interaction Complexity (IC)	0.15	0.00	3	7.13
Click Asymmetry (CA)	−0.004	0.00	2	13.30
Effort Engineering Score	0.163	0.111	0.889	100.00

**Table 9:** Cognitive Load Amplification metrics derived from textual representations.

Metric	Mean	Median	Max	Non-Zero (%)
Word Count	166.82	0.00	2446	23.99
Information Density (1–5)	1.40	1.00	5	100.00
Semantic Ambiguity Hits	—	—	85	—
Disclosure Complexity Hits	1.04	0.00	153	10.31
Cognitive Load Score	0.044	0.000	0.655	23.99

**Table 10:** Consent-state complexity indicators derived from preference system reconstruction.

Metric	Mean	Median	Max	Non-Zero (%)
Toggle Count	1.03	0.00	2465	4.13
Vendor Keyword Hits	0.31	0.00	120	4.10
Category Keyword Hits	0.70	0.00	353	4.38
Disclosure Complexity Hits	1.04	0.00	153	10.31

**Table 11:** Consent-state verification and complexity metrics.

**Panel A. Consent-State Verification Outcomes**			
**Metric**	**Frequency**	**Percentage (%)**	
Consent-State Observable	2159	11.57	
Third-Party Tracking Evidence	3046	16.32	
Storage Artifact Evidence Present	1154	6.18	
Network Tracking Evidence Present	1416	7.59	
**Panel B. Consent-State Complexity Statistics**			
**Metric**	**Mean**	**Median**	**Max**
Storage Artifact Evidence Hits	0.84	0.00	134
Network Tracking Evidence Hits	0.46	0.00	66
Consent-State Complexity Score	0.045	0.000	0.632

**Table 12:** ACL-DP composite mechanism scores.

Composite Metric	Mean	Median	Max
Effort Engineering Score	0.163	0.111	0.889
Attention Engineering Score	0.016	0.000	1.000
Cognitive Load Score	0.044	0.000	0.655
Consent-State Complexity Score	0.045	0.000	0.632
ACL-DP Total Score	0.268	0.111	2.464

**Table 13:** Execution outcomes of the consent-state verification layer.

Execution Outcome	Frequency	Percentage (%)
SUCCESS	1951	19.51
STAGE5_FAIL	7116	71.17
NAVIGATION_FAIL	819	8.19
TIMEOUT	112	1.12
Total	9998	100.00

**Table 14:** Consent mismatch detection results with 95% confidence intervals.

Consent Mismatch Metric	Frequency	Percentage (%)	95% CI (%)
Records with Measurable CM Evaluation	161	100.00	–
Consent Mismatch Detected (CM=1)	125	77.64	[70.47, 83.47]
Synchronization Consistent (CM=0)	36	22.36	[16.53, 29.53]

Note: Consent mismatch evaluation was restricted to environments containing sufficient synchronized frontend and backend evidence for direct verification of consent-state propagation behavior.

**Table 15:** Distribution of ACL-DP mechanism family scores.

Mechanism Family	Mean	Median	95th Percentile	Max
Effort Engineering (EE)	0.157	0.100	0.653	0.900
Attention Engineering (AE)	0.046	0.000	0.260	0.841
Cognitive Load Amplification (CL)	0.036	0.000	0.206	0.603
Algorithmic Adaptivity (AA)	0.029	0.000	0.201	0.708
ACL-DP Total Score	0.067	0.025	0.289	0.615
ACL-DP Total Score (0–100)	6.72	2.50	28.92	61.50

**Table 16:** Distribution of ACL-DP intensity levels.

ACL-DP Intensity	Frequency	Percentage (%)
Low	9329	93.29
Moderate	665	6.65
High	6	0.06
Very High	0	0.00

**Table 17:** Top-ranked domains according to ACL-DP composite scores.

Domain	EE	AE	CL	AA	ACL-DP Score	ACL-DP (0–100)
newsweek.com	0.656	0.790	0.348	0.666	0.615	61.50
jampp.com	0.756	0.530	0.378	0.511	0.544	54.37
statista.com	0.663	0.788	0.387	0.200	0.510	50.95
altervista.org	0.658	0.841	0.404	0.133	0.509	50.90
ipinfo.io	0.654	0.821	0.333	0.203	0.503	50.28
rightmove.co.uk	0.360	0.543	0.453	0.644	0.500	50.00
flashscore.com	0.360	0.586	0.396	0.640	0.496	49.56
msn.com	0.353	0.551	0.430	0.644	0.494	49.44
gumgum.com	0.658	0.782	0.362	0.167	0.492	49.20
blackberry.net	0.656	0.782	0.286	0.202	0.482	48.15

**Table 18:** Associations between ACL-DP mechanism families and the composite ACL-DP score.

Association	Spearman’s (ρ)	FDR-Adjusted (*p*)	Significant
EE → ACL-DP Total	0.922	0.000	Yes
AE → ACL-DP Total	0.894	0.000	Yes
CL → ACL-DP Total	0.931	0.000	Yes
AA → ACL-DP Total	0.656	0.000	Yes

**Table 19:** Inter-mechanism associations among ACL-DP mechanism families.

Association	Spearman’s (ρ)	FDR-Adjusted (*p*)
EE–CL	0.970	0.000
AE–CL	0.950	0.000
EE–AE	0.895	0.000
EE–AA	0.452	0.000
AE–AA	0.467	0.000
CL–AA	0.472	0.000

**Table 20:** Comparison of existing computational approaches for consent ecosystem analysis.

Capability	CMP Audits	Browser Crawlers	Dark-Pattern Studies	ACL-DP
CMP/banner detection	Full [7,8]	Full [20]	Partial [21]	Full
Dynamic interaction reconstruction	Limited [7]	Partial [2,12]	Limited [21]	Full
Frontend–backend synchronization	None [8]	None [20]	None [22]	Full
Consent-state verification	Limited [8]	Partial [2,3]	None [21]	Full
Adaptive manipulation scoring	None [7]	None [23]	Partial [5,22]	Full
Human validation benchmark	Partial [8]	Rare [12]	Partial [5,21]	Full

*Note:* **Full** indicates comprehensive support for the capability; **Partial** indicates that the capability is supported only to a limited extent or under specific conditions; **Limited** indicates minimal or restricted support; **Rare** indicates that the capability has been reported only in a small number of studies; and **None** indicates that the capability is not supported.

## Data Availability

The original contributions presented in this study are included in the article.
